# Exploring therapeutic architectural strategies as recovery- supportive design interventions in selected international sanatorium and therapeutic wellness facilities

**DOI:** 10.3389/fpsyg.2026.1830779

**Published:** 2026-06-25

**Authors:** Eghosa N. Ekhaese, Bukola T. Salami

**Affiliations:** Department of Architecture, Covenant University, Ota, Nigeria

**Keywords:** evidence-based design, holistic care, observational assessment, patient recovery, sanatorium design, therapeutic architectural elements, therapeutic architectural strategies, therapeutic facilities

## Abstract

**Background:**

Sanatoriums, initially developed for the treatment of tuberculosis, emphasise therapeutic architecture strategies that integrate design elements that enhance patient well-being and recovery. However, modern healthcare facilities often overlook these strategies, resulting in environments that prioritise illness over holistic care. The application of therapeutic architectural strategies (TASs), such as biophilic design, lighting design, and the integration of outdoor spaces, can create restorative therapeutic settings. Despite their benefits, challenges such as inadequate implementation, environmental exposure, climate sensitivity, and accessibility limit their effectiveness.

**Purpose:**

This study explores the presence, quality, and recovery-supportive associations of TASs within selected international sanatorium and wellness facilities, using structured observational methods and literature-derived proxy recovery indicators to identify key therapeutic architectural elements (TAEs) that contribute to patient well-being.

**Methods:**

A mixed-methods approach was adopted, employing structured observational checklists applied to publicly available architectural documentation, professional photography, and academic literature across three internationally recognised facilities. Spearman’s rank correlation was used to examine the association between TAS implementation scores and proxy recovery indicator scores.

**Results:**

The findings indicate that TASs such as privacy and personalisation, colour psychology, and material selection are strongly associated with recovery-supportive design conditions, consistent with outcomes documented in the therapeutic architecture literature. A strong positive association (*ρ* = 0.83) was observed between TAS implementation scores and proxy recovery indicator scores; however, inadequate implementation and climate sensitivity pose contextual challenges.

**Conclusion:**

The study highlights the need for evidence-based design (EBD) principles in therapeutic architecture and presents an observational assessment framework for integrating TASs into sanatorium and wellness facility design. The findings offer associative evidence supporting recovery-supportive design practice, with particular relevance to under-resourced healthcare contexts such as Nigeria.

## 1 Introduction

The design of therapeutic facilities has undergone a significant evolution in recent decades, moving beyond conventional medical efficiency to embrace holistic healing environments that support patient well-being (Feng et al., 2024; Liang et al., 2025). This shift is grounded in the growing understanding that the built environment can serve as a powerful agent in promoting physical, psychological, and emotional recovery (Rebecchi et al., 2023; Wilson et al., 2023). In this context, therapeutic architecture has emerged as a critical approach to healthcare design, advocating for spatial strategies that actively support healing and the overall patient experience (Ghalehnoei et al., 2022). Among the most impactful therapeutic strategies are biophilic design, which fosters connection to nature; lighting design, which regulates circadian rhythms; and acoustic design, which minimises stress-inducing noise (Antoniadou et al., 2025; Suresh et al., 2025). Similarly, spatial organisation ensures functional efficiency and psychological clarity, while integrating outdoor spaces creates restorative environments that promote calm and rejuvenation (Dong et al., 2024; Wang et al., 2024; Akinola and Ini-Ukim, 2025). Ekhaese and Ezeora (2024) highlighted the role of TASs in a rehabilitation centre for alcohol and drug sufferers in Nigeria. They found that features such as privacy, acoustic comfort, and natural environments improve recovery, underscoring the importance of integrating psychosocial needs into architectural design. According to Finnigan (2024), Malhotra and Abrol (2024), Gao and Zhu (2025) and Pawlaczyk-Szymańska et al. (2025), other essential elements include privacy and personalisation, which empower patient autonomy; colour psychology and material selection, which affect mood and perception; adaptive spaces that respond to varying user needs; and sensory integration, which engages multiple senses for a holistic healing experience. Despite their documented benefits, these strategies are often inadequately implemented in therapeutic environments, particularly in developing contexts where facility design remains primarily utilitarian (Jaušovec and Gabrovec, 2023; Alhmoud, 2024). This shortfall can lead to environments that prioritise disease management over comprehensive recovery, undermining the broader goals of patient-centred care (Levine and Drossman, 2022; Martyushev-Poklad et al., 2022; Vareta et al., 2025). Moreover, challenges such as climate sensitivity, infrastructure limitations, and limited awareness further constrain the application of TASs, especially in sub-Saharan Africa (Nyatuka and De La Harpe, 2022; Mba et al., 2024; Atewologun et al., 2025). In Nigeria, the need to adopt TASs is increasingly urgent. Sanatoriums and long-term therapeutic care centers are often overstretched, under-optimized, and lacking in patient-centered design (PCD) features (Goldmann et al., 2023; Boamah et al., 2025). Sholanke and Oke (2020) evaluated therapeutic landscapes in medical facilities in Ota, Nigeria. Their findings showed partial adoption of healing gardens and green spaces, with potential to reduce stress and improve recovery if systematically implemented.

This study explores TASs in selected international sanatorium and wellness facilities and their association with recovery-supportive design conditions, specifically by identifying TAEs that contribute to patient well-being and examining how TAS implementation corresponds with proxy recovery indicators. Despite growing recognition of therapeutic architecture’s role in patient recovery, there remains a significant gap in structured, comparative assessments of TAS implementation across international therapeutic facilities and their implications for under-resourced contexts such as Nigeria. This study addresses that gap through two research questions: (1) What TASs are present and to what quality level are they implemented across the selected facilities? (2) What is the association between TAS implementation scores and proxy recovery indicator scores? The international case studies serve as aspirational benchmarks from which transferable TAS principles can be extracted and adapted to the Nigerian healthcare design context, where facilities often prioritise clinical efficiency over therapeutic impact (Ogah et al., 2024; Mba et al., 2024). These insights are particularly relevant to contexts such as Nigeria, where evidence-based design adoption remains limited. The data can assist architects and healthcare providers in prioritizing features such as access to nature, optimized lighting and acoustics, and comfortable social spaces when designing recovery-supportive therapeutic wellness facilities.

## 2 Literature review

Therapeutic architecture, often referred to as salutogenic design or generative space, has garnered global recognition in recent years for its role in creating built environments that actively contribute to healing and well-being (Rakhshani and Khakzand, 2025). Yang (2023) describes therapeutic architecture as a people-centered, evidence-based discipline of the built environment that incorporates spatial elements that engage users physiologically and psychologically to support recovery. By integrating design components such as natural ventilation, acoustic comfort, and visual and spatial aesthetics, therapeutic architecture is strongly associated with enhanced patient well-being and improved recovery-supportive conditions (Cho, 2023; Faraj et al., 2024). However, while these benefits are widely documented in high-income healthcare settings, their translation to under-resourced environments such as those in sub-Saharan Africa remains insufficiently examined, representing a critical gap this study addresses (Nyatuka and De La Harpe, 2022; Ogah et al., 2024). The origins of therapeutic architecture can be traced to the development of sanatoriums in the late 19th and early 20th centuries (Collins, 2020). These early healthcare facilities, built to treat chronic illnesses like tuberculosis, were typically situated in serene natural environments, mountainous regions, coastal areas, or forested landscapes selected for their fresh air, sunlight, and tranquil settings (Avci-Hosanli and Degirmencioglu, 2024; Tate et al., 2024).

According to Sal Moslehian et al. (2023) and Yan and Geng (2024), the architectural features of these sanatoriums embodied design strategies that leveraged the healing potential of nature, emphasising open terraces, operable windows, and south-facing orientations to maximise daylight and ventilation. Babalola et al. (2024) designed a therapeutic student centre for Covenant University using a user-centred approach. Their study showed that strategies such as biophilic design, adaptive spaces, and material selection enhance psychosocial well-being, proving therapeutic architecture’s relevance beyond healthcare into educational settings. Therapeutic architecture thus represents a shift from conventional, illness-focused healthcare design toward a holistic, person-centered approach. Holt-Lunstad et al. (2025) and Massey et al. (2025), identify a set of nine core principles central to TASs: Biophilic Design, Lighting Design, Acoustic Design, Spatial Organisation, Integration of Outdoor Spaces, Privacy and Personalisation, Colour Psychology and Material Selection, Adaptive Spaces, and Sensory Integration. Each of these strategies contributes to healing by addressing patients’ sensory, cognitive, and emotional needs. Within the Evidence-based design (EBD) framework, these nine strategies collectively address three domains of patient health outcomes, physiological (e.g., circadian regulation, pain reduction), psychological (e.g., stress reduction, mood improvement), and behavioural (e.g., outdoor engagement, social interaction) providing a structured basis for evaluating their recovery-supportive potential (Cho, 2023; Simonsen et al., 2022; Li et al., 2024). Biophilic design, for example, reconnects users with nature through elements such as greenery, natural light, and water features, which have been linked to reduced stress and faster recovery (Cacique and Ou, 2022; Tekin et al., 2025). Lighting design improves circadian regulation and mood, while acoustic design ensures sound comfort and reduces stress-inducing noise levels (Bayır, 2024).

Read and Meath (2025) explained that the evolution of TASs closely aligns with the broader discipline of EBD, which uses empirical data and research to inform design decisions. Within EBD, therapeutic spaces incorporate scientifically supported architectural elements, such as access to daylight, ventilation, outdoor views, ergonomic layouts, and calming materials, to support healing (McDonald et al., 2024; Sabet et al., 2025; Zhu et al., 2025). David-mukoro et al. (2024) evaluated therapeutic landscapes in medical facilities in Nigeria. Their findings showed partial adoption of healing gardens and green spaces, with potential to reduce stress and improve recovery if systematically implemented. This partial adoption reflects a broader pattern identified across developing contexts, where design priorities remain primarily utilitarian rather than therapeutic (Jaušovec and Gabrovec, 2023). Taken together, these studies underscore the need for a structured, comparative framework for assessing TAS implementation which this study provides. These principles are especially pertinent in long-term care environments like sanatoriums, where patients benefit from restorative design features integrated throughout their daily experience (Li et al., 2025). In therapeutic architecture, design is not just about creating functional healthcare infrastructure; it is about shaping experiences that support comprehensive recovery (Simonsen et al., 2022). Through the integration of multisensory design, personalised spaces, and nature-oriented elements, TASs transform healthcare environments into nurturing settings that respond to the diverse needs of their users (Liu et al., 2021; Ebaid, 2023). This approach holds particular significance in the Nigerian context, where healthcare facilities often prioritise clinical efficiency over therapeutic impact, underscoring a growing need for architectural solutions aligned with global best practices in healing environments (Ogah et al., 2024).

## 3 Materials and methods

This study explores the implementation of TASs across selected international sanatorium and wellness facilities and their association with recovery-supportive design conditions. A case study research method was adopted to provide an in-depth, contextualised analysis of how these strategies are applied across diverse geographic and climatic settings. It is important to note that this study does not collect primary clinical or patient-level data. Rather, it adopts an Evidence-Based Design (EBD) observational approach, evaluating the presence and quality of architectural features and mapping them to proxy recovery indicators validated in the existing literature (Rebecchi et al., 2023; Li et al., 2024). The findings therefore reflect the recovery-supportive potential of the built environment rather than directly measured clinical outcomes.

### 3.1 Research design

This study adopted a case study design, using a structured observation guide and correlation analysis to evaluate the implementation of TASs across three healthcare facilities: Chiva-Som Health Resort, Thailand (CSHR); MAYRLIFE Medical Health Resort, Austria (MMHR); and The Chedi Andermatt, Switzerland (TCA). This study is guided by two research questions: (1) What TASs are present and to what quality level are they implemented across the selected international sanatorium and wellness facilities? (2) What is the association between TAS implementation scores and proxy recovery indicator scores across the selected facilities?. This design enabled a systematic comparison of architectural elements across differing geographic, climatic, and cultural contexts. The approach aligns with EBD practices commonly used in healthcare architecture research (Van der Zwart and Pilosof, 2020; Oher et al., 2024).

### 3.2 Sampling technique

A purposive sampling technique was used to select the three case studies. Facilities were selected based on their global recognition, reputation for wellness-oriented architecture, and published documentation of therapeutic outcomes. Purposive sampling allows for the deliberate selection of facilities that are information-rich and relevant to the research objectives (Dahal et al., 2024). The selected facilities were required to meet the following criteria: Internationally recognised for wellness and therapeutic design, operate as an internationally recognised sanatorium, health resort, or therapeutic wellness facility, have accessible visual, architectural, and/or academic documentation and integrate known TASs (e.g., biophilic design, sensory integration). These criteria ensured that the facilities analysed had sufficient design complexity and relevance to therapeutic architecture. Therefore, the facilities were selected based on: Location in diverse climatic zones (tropical, alpine, temperate), variety in therapeutic services offered (e.g., spa, wellness medicine, rehabilitation), presence of published research or professional analysis on their TASs and availability of floor plans, virtual tours, or high-resolution documentation for structured observation.

To evaluate the presence and quality of TASs, a structured observation guide was developed based on a scoring matrix derived from literature (Rebecchi et al., 2023; Li et al., 2024). Nine core strategies were assessed: *Biophilic Design, Lighting Design, Acoustic Design, Spatial Organisation, Integration of Outdoor Spaces, Privacy and Personalisation, Colour Psychology and Material Selection, Adaptive Spaces, and Sensory Integration*. Observation was conducted using publicly available architectural documents, official resort websites, published academic analyses, a qualitative production checklist, and professional photography from peer-reviewed design sources.

### 3.3 Data collection

The data collection instruments employed for this study were a structured observational checklist developed from a literature review of TASs and a qualitative photo-production checklist. It should be clarified that the term “questionnaire” as used in earlier versions of this study refers to the structured observational checklist items applied by the researchers during systematic observation, these are evaluative criteria scored by the research team, not patient-administered surveys. No primary patient data was collected. The checklist focused on nine TASs. Given the comprehensive nature of the observational checklist, Tables 1, 2 present detailed feature-level data across all nine TAS categories. While extensive, this level of detail is necessary to ensure transparency and reproducibility of the observational assessment. Summary scores are consolidated in Table 3 for ease of interpretation. Structured observation sheets were used to score each facility based on visual and spatial features visible in architectural images, virtual tours, official descriptions, and verified academic publications. Additional data were drawn from scientific literature on each facility’s therapeutic outcomes, user reviews, and professional design evaluations. This combination of visual data and literature-based health metrics facilitated robust comparative evaluation. Table 4 presents the selected facilities chosen as case studies for this research.

**Table 1:** Implemented TASs in the selected facilities.

S/N	Therapeutic architectural strategies Scale of availability where A-(Available) indicates presence, and N/A-(Not Available) indicates absence of TASs in the facility.		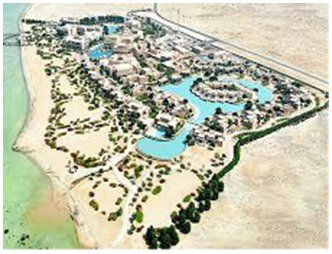		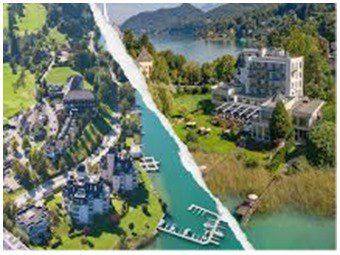		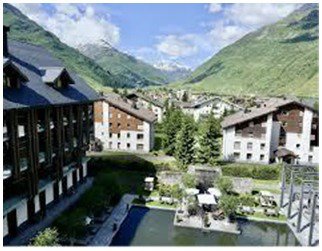	
			CSHR, Thailand		MMHR, Austria		TCA, Switzerland	
			A	N/A	A	N/A	A	N/A
1	Biophilic Design	Indoor Gardens and Green Spaces	✓		✓		✓	
		Water Features	✓		✓		✓	
		Healing Gardens	✓		✓			✓
		Rooftop Terraces and Green Roofs		✓		✓		✓
		Healing Lounges and Social Hubs	✓		✓		✓	
2	Lighting Design	Natural Lighting	✓		✓		✓	
		Therapeutic Colored Lighting	✓		✓		✓	
		Lighting Controls	✓		✓		✓	
3	Acoustic Design	Library and Reading Rooms	✓		✓		✓	
		Spiritual and Reflection Rooms		✓		✓		✓
		Meditation and Relaxation areas	✓		✓		✓	
4	Spatial Organisation	Signages	✓		✓		✓	
		Pavilions and Gazebo	✓					
		Dining and Nutrition Spaces	✓		✓		✓	
		Outdoor Seating Zones	✓		✓		✓	
5	Integration of Outdoor Spaces	Outdoor Therapy Areas	✓		✓			✓
		Open-Air Yoga Areas	✓		✓		✓	
		Walking Trails and Jogging Paths	✓		✓		✓	
6	Privacy and Personalisation	Patient Rooms	✓		✓		✓	
		Hydrotherapy Rooms		✓		✓	✓	
		Therapy Rooms	✓		✓		✓	
		Rehabilitation Gym	✓		✓		✓	
7	Colour Psychology and Material Selection	Soothing Colours- Blues/Greens	✓		✓		✓	
		Earthy Tones and Warm Colours- Muted Oranges and Yellows	✓			✓		✓
		Neutral and Pastel shades- Off-white, beige and pastels		✓	✓		✓	
		Wood	✓					
		Stone	✓			✓		✓
		Bamboo	✓			✓		✓
8	Adaptive Spaces	Multifunctional Rooms		✓	✓			✓
		Nature-Integrated Areas	✓			✓	✓	
9	Sensory Integration	Aromatherapy	✓		✓		✓	
		Soft Furnishings	✓		✓		✓	
		Sensory Rooms		✓	✓			✓
		Total	27		25		23	

**Table 2:** Quality assessment of TAS across selected facilities.

S/N	Therapeutic Architectural Strategies Likert Scale of Quality, which indicates 1-Poor, 2- Fair, 3- Good, 4- Very Good, 5- Excellent	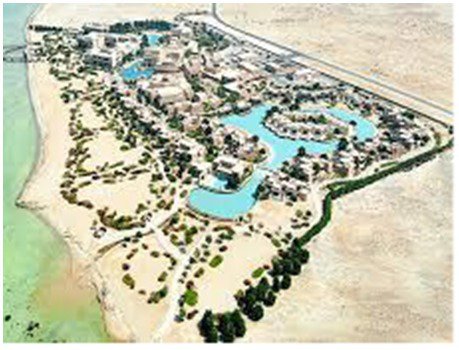					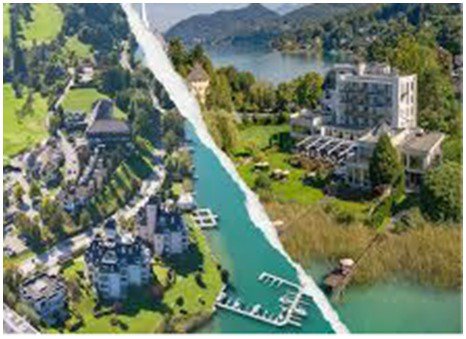					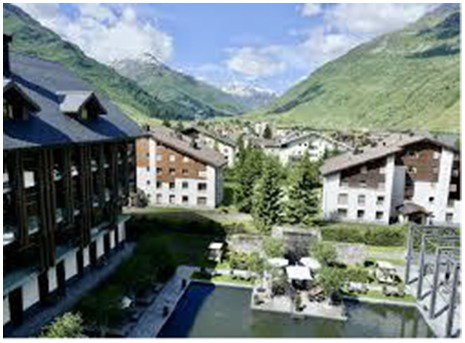					Remarks
		CSHR, Thailand					MMHR, Austria					TCA, Switzerland					
		1	2	3	4	5	1	2	3	4	5	1	2	3	4	5	
Strategy 1: Biophilic Design																	CSHR, Thailand, leads with lush gardens and beachfront integration; MMHR, Austria, uses mountain views; TCA, Switzerland, relies on alpine scenery but less immersive greenery.
1	Large windows				✓					✓				✓			
	Skylights			✓						✓			✓				
	Indoor plants				✓				✓				✓				
	Green walls				✓			✓				✓					
	Water Features					✓			✓				✓				
	Views of Nature				✓					✓				✓			
Total		24					20					13					
Category 2: Lighting Design																	CSHR, Thailand, maximises natural and ambient light; MMHR, Austria, emphasises daylight with reflective interiors; TCA, Switzerland, uses atriums and smart LED systems.
2	Smart Lighting				✓					✓				✓			
	Light wells		✓						✓				✓				
	Solar Shading Devices				✓				✓					✓			
Total		34					30					21					
Strategy 3: Acoustic Elements																	CSHR, Thailand, employs water features and sound-absorption; MMHR, Austria, offers quiet lounges; TCA, Switzerland, relies on fireplaces and soft furnishings.
3	Sound-absorbing materials			✓					✓				✓				
	Quiet zones				✓					✓			✓				
	Noise-reducing mechanisms		✓						✓				✓				
Total		43					40					27					
Strategy 4: Spatial Organisation Elements																	CSHR, Thailand, prioritises privacy with clear zoning; MMHR, Austria, ensures open, connected layouts; TCA, Switzerland, emphasises communal open-plan spaces.
4	Intuitive layouts			✓					✓					✓			
	Clear Wayfinding			✓					✓					✓			
	Centralised communal spaces				✓					✓				✓			
Total		53					50					36					
Strategy 5: Integration of Outdoor Spaces																	
5	Balconies			✓					✓					✓			CSHR, Thailand, excels with beachfront gardens and pavilions; MMHR, Austria, blends terraces with mountain views; TCA, Switzerland, offers private terraces but limited therapy gardens.
	Garden				✓					✓				✓			
	Covered walkways	✓						✓					✓				
Total		61					59					44					
Strategy 6: Privacy and Personalisation Elements																	CSHR, Thailand, provides personalised treatment rooms; MMHR, Austria, offers flexible accommodation options; TCA, Switzerland, ensures privacy with customisable luxury rooms.
6	Private rooms				✓					✓				✓			
	Adjustable furnishings				✓				✓					✓			
Total		69					66					50					
Strategy 7: Colour Psychology and Material Selection Elements																	CSHR, Thailand, uses calming natural palettes; MMHR, Austria, blends traditional wood and stone; TCA, Switzerland, adopts warm, earthy chalet aesthetics.
7	Calming colour palettes,				✓				✓						✓		
	Natural materials				✓					✓						✓	
Total		77					73					59					
Strategy 8: Adaptive Spaces elements																	CSHR, Thailand, uses modular layouts; MMHR, Austria, promotes multipurpose spaces; TCA, Switzerland, integrates multifunctional rooms with some fixed limits.
8	Modular furniture			✓				✓									
	Flexible layouts			✓					✓								
	Movable partitions			✓				✓							✓		
Total		86					80					69					
Strategy 9: Sensory Integration elements																	CSHR, Thailand, engages multiple senses through scents, textures, and natural stimuli; MMHR, Austria, uses tactile and scented environments; TCA, Switzerland, emphasises spa-centred aromatherapy.
9	Touch-sensitive materials	✓					✓					✓					
	Diffusion systems		✓					✓						✓			
	Scent zones			✓							✓						
Total		96					91					81					

**Table 3:** Comparative analysis of selected facilities.

Therapeutic architectural strategies (TASs)	International sanatorium and wellness facilities		
	CSHR, Thailand	TCA, Switzerland	MMHR, Austria
	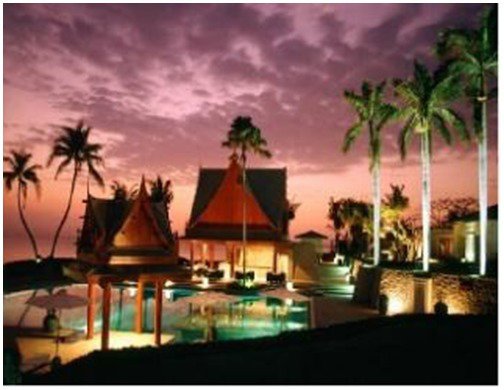	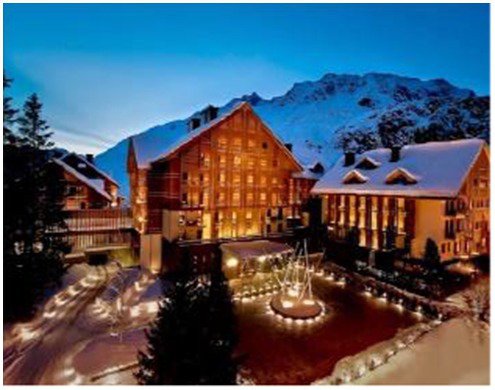	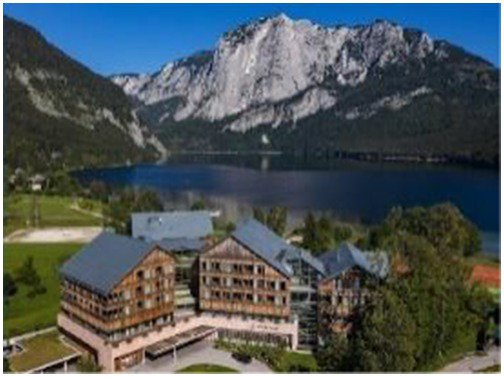
	Score		
Implemented TASs in selected facilities	27	23	25
Quality Assessment of TASs across facilities	96	81	91
Cumulative Score	123/171	104/171	116/171
Percentage %	71.93	60.82	67.25
Ranking	1st	3rd	2nd

Source: author’s fieldwork 2025.

**Table 4:** List of selected international sanatorium facilities and wellness facilities.

S/N	Sanatoriums and wellness facilities	Location
1	Chiva-Som Health Resort (CSHR)	Thailand
2	MAYRLIFE Medical Health Resort (MMHR)	Austria
3	The Chedi Andermatt Wellness Facility (TCA)	Switzerland

These include the CSHR in Hua Hin, Thailand, recognised globally for its integrative approach to wellness and therapeutic design; the MMHR in Altaussee, Austria, which combines medical expertise with restorative architectural features; and TCA in Switzerland, a luxury wellness facility that integrates TASs within a mountainous environment. The selection of these facilities provides a diverse geographical and cultural context, enabling comparative insights into how TASs are applied across different international settings.

### 3.4 Data analysis

The quantitative data were analysed using Spearman’s rank correlation to examine the association between TAS implementation scores and proxy recovery indicator scores across the three selected facilities. Given the exploratory nature of this study and the small number of facilities assessed (*n =* 3), the Spearman’s rank correlation is applied here as an indicative analytical tool rather than a basis for broad inferential conclusions. The scoring framework was developed from a structured review of peer-reviewed therapeutic architecture literature (Rebecchi et al., 2023; Li et al., 2024), with checklist criteria grounded in established EBD assessment practice. Future studies should incorporate inter-rater reliability testing and formal validation of the scoring framework against established clinical or architectural assessment tools. The average score for each strategy per facility was compared with available patient-centred outcomes, such as recovery-focused services, wellness success rates, and user well-being. The analysis helped establish statistical relationships between TAEs and therapeutic outcomes, offering insight into their effectiveness across diverse contexts (Boemo et al., 2022).

Qualitative data were analysed using *a priori* thematic analysis. The researchers employed the TAEs checklist for the primary data analysis. Observational notes were developed and analysed using thematic analysis across the nine TASs. The authors deductively interpreted under a pre-existing theme: TAEs available in case studies; TAEs in selected healthcare facilities that enhance patients’ well-being; TAEs in selected healthcare facilities that promote patient recovery; and the adoption of TASs across selected healthcare facilities. The authors presented the results using tables, figures, images, graphs, and charts.

## 4 Results and findings

Therapeutic architecture is a PCD and EBD approach that uses architectural elements to support the physiological and psychological well-being of building users (Bolpagni et al., 2024). Bulaj et al. (2025) the results are presented in alignment with the two research questions: (1) identifying TAEs in selected international sanatorium and wellness facilities that contribute to patient well-being, and (2) examining the association between TAS implementation and proxy recovery indicator scores across the selected facilities. The assessment technique for quantitative and qualitative data depends on objectives, methodology, and analysis. However, the authors presented results chronologically, aligned with the objectives, and used tables, charts, figures, screen plots, and images.

### 4.1 TAEs in selected international sanatorium and wellness facilities that enhance patients’ well-being

**Figure 1:**
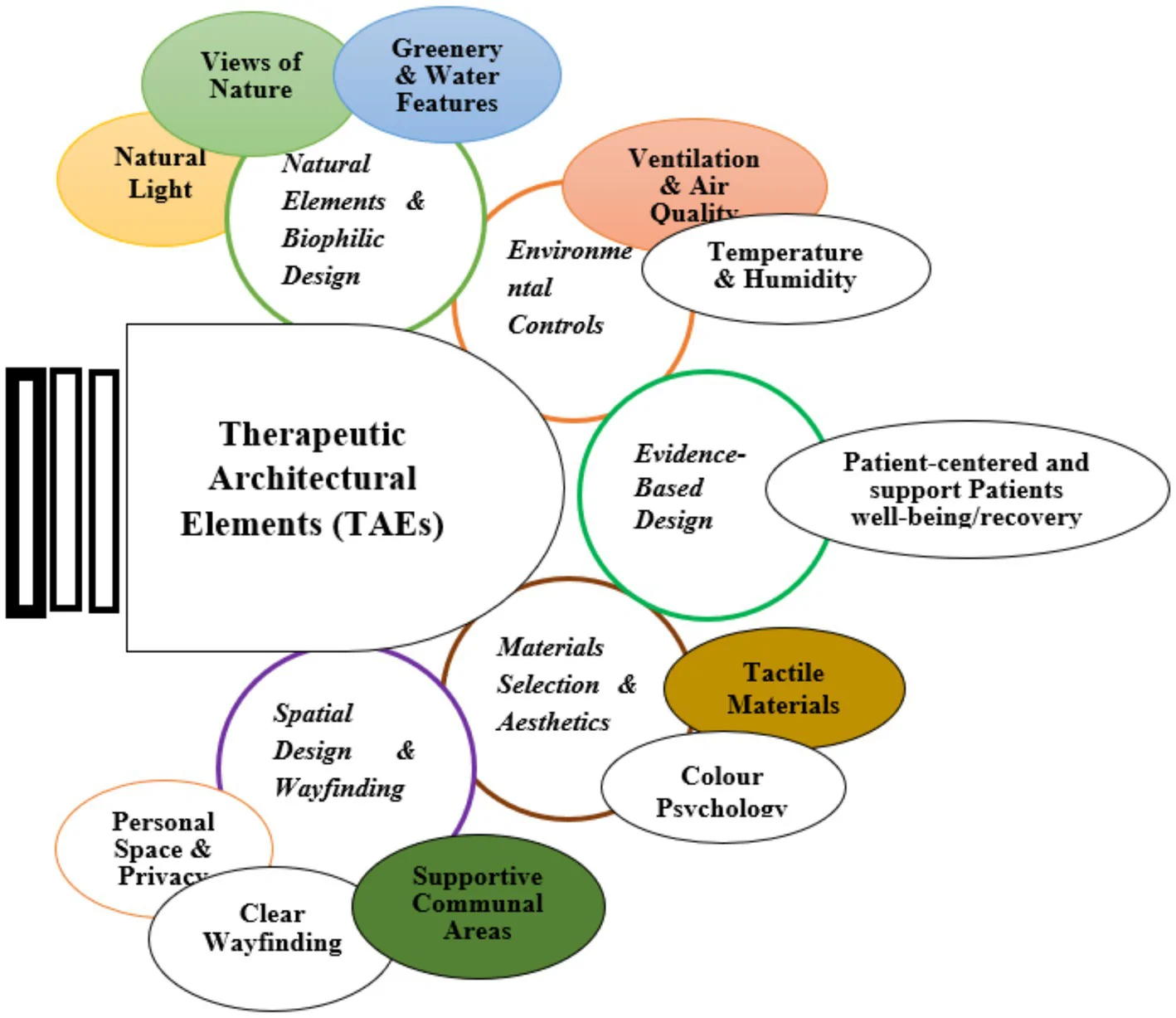
TAEs in healthcare facilities that enhance patient well-being and recovery; source: Author (2025), adapted from Nair (2022).

Figure 1 presents the TAEs of a health resort checklist as: Medicals (treatments), Spa and Beauty (Bath, beauty, care massage), Fitness (gym, yoga studios, hydrotherapy), Sports (tennis court, swimming, golf course, sky), Nature (Parks, garden, lakes, pool, ocean access), Education (aquarium, Training hall, museum, classrooms), Community (heritage, social connectedness), Nutrition (healthy local food) that guide the photoproduction process. Three markers drive the essential TAEs of a health resort- 1. Pleasure and Well-being (medicals, spa, beauty and Fitness), 2. Healing experience (Sports, nature and education) and 3 Community engagement (Nutrition, Community and education).

The well-being benefits derived from using TAEs in a healthcare facility from literature and corroborated by professionals include *improved patient recovery, reduced stress, anxiety, and pain, enhanced mental health and cognitive, comfort, improved mood and wellness, improve air quality, fostering a calming, supportive, and patient-centred environment and better QoL* (Nair, 2022; Cho, 2023; Faraj et al., 2024; Akinola and Ini-Ukim, 2025).

**Figure 2:**
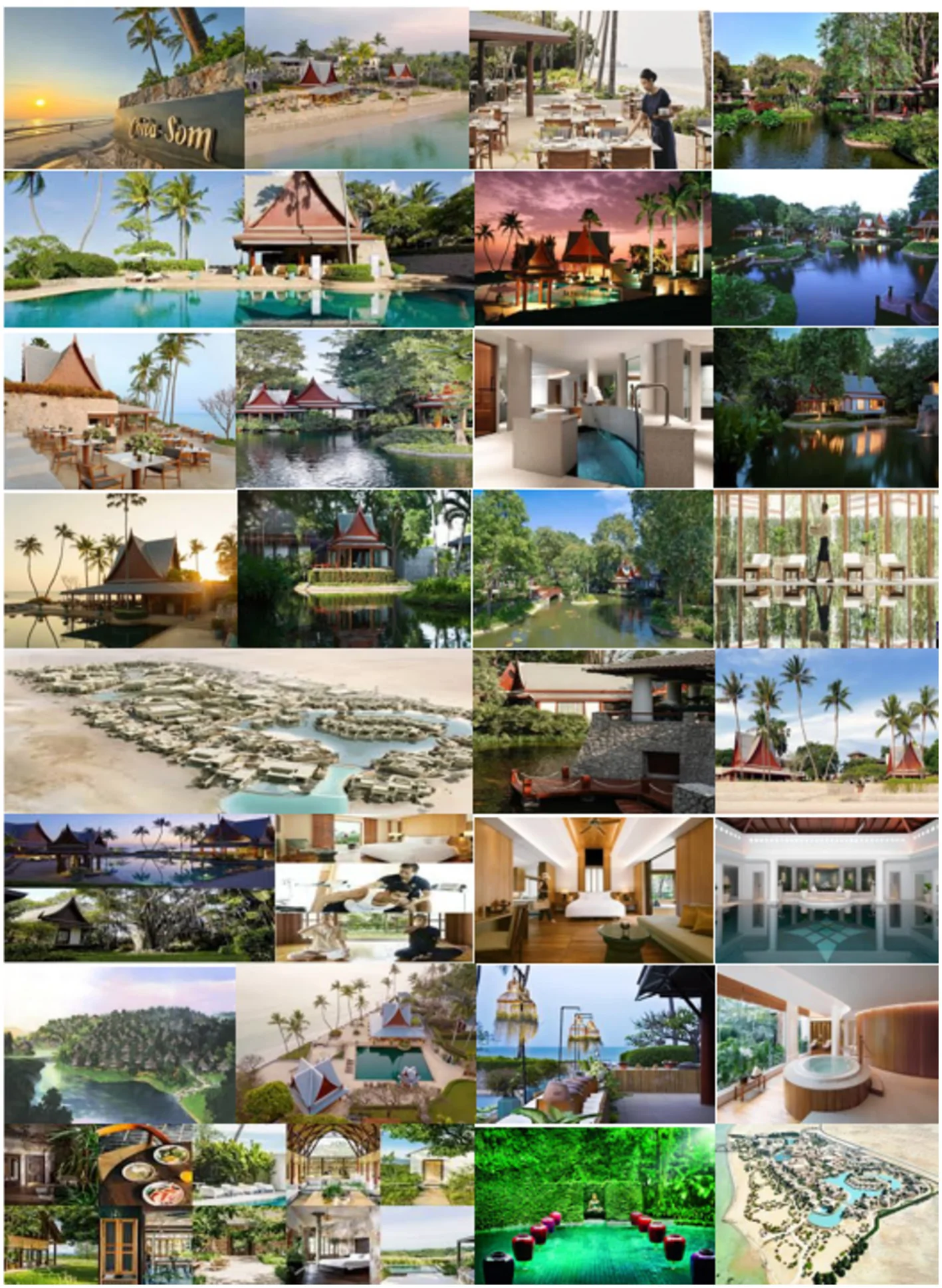
Qualitative Photoproduction of Chiva-Som Health Resort, Hua Hin, Thailand.

**Figure 3:**
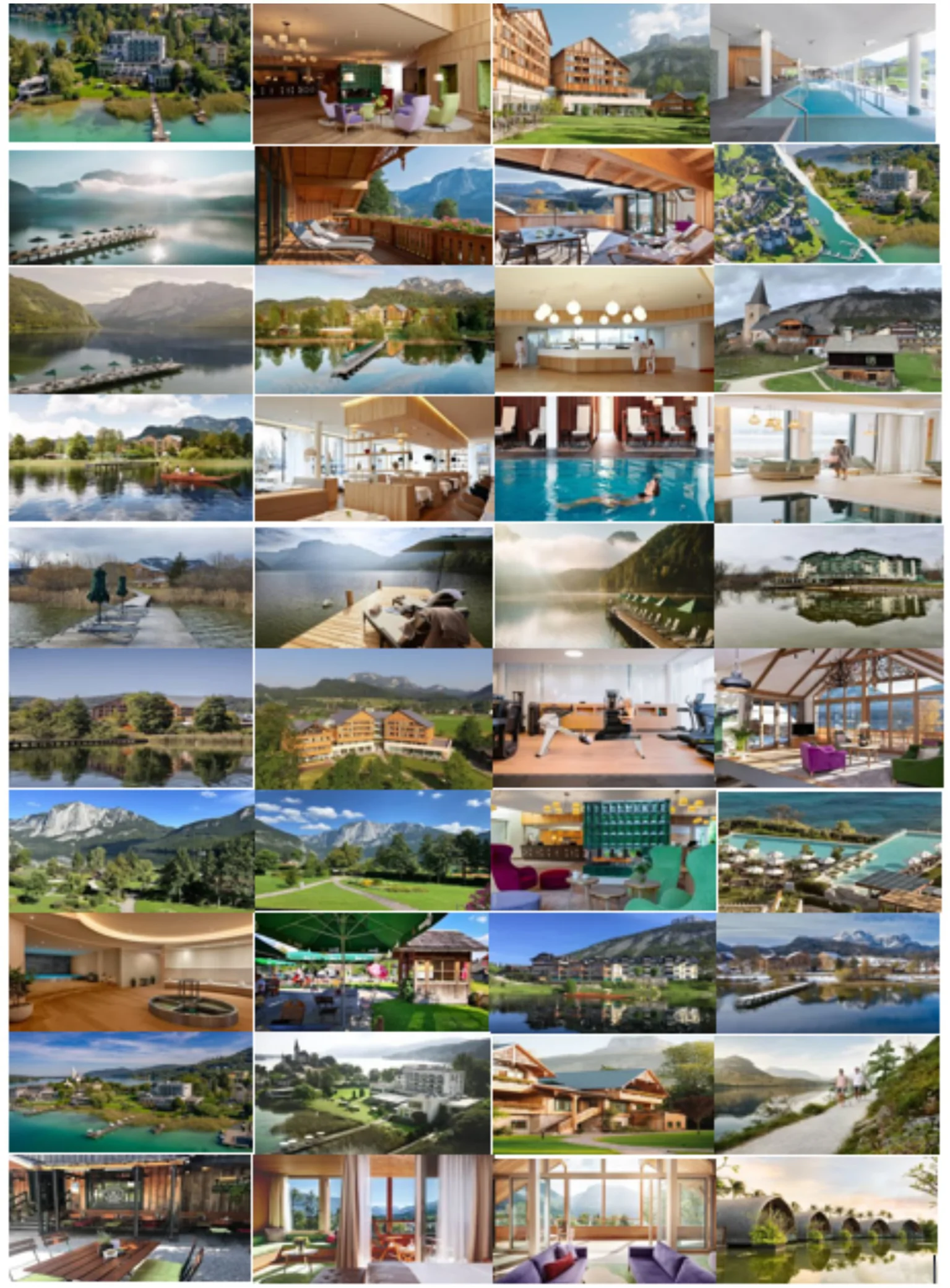
Qualitative Photoproduction of Mayrlife Medical Health Resort, Altaussee, Austria.

**Figure 4:**
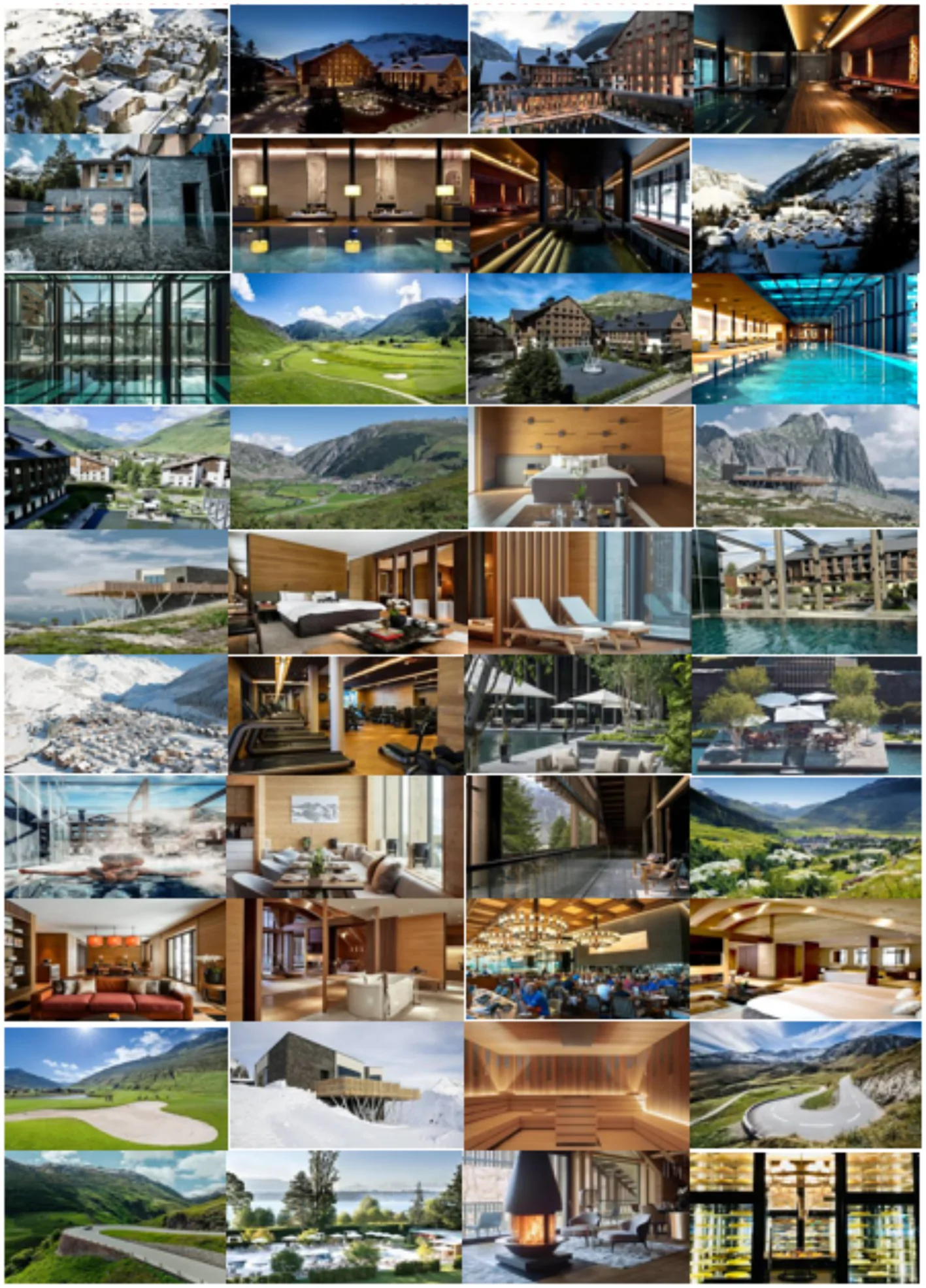
Qualitative Photoproduction of The Chedi Andermatt, Wellness Facility, Gotthardstrasse, Andermatt, Switzerland.

The authors conducted a qualitative photo- production checklist analysis and reached saturation with the three selected international facilities. A total of 30 photographs sourced from publicly available architectural documentation and professional photography were analysed for case study 1-CSHR, Hua Hin, Thailand, as shown in Figure 2. A total of 40 photographs were similarly sourced and analysed for case study 2-MMHR, Altaussee, Austria, as shown in Figure 3, and 40 for case study 3-TCA, Gotthardstrasse, Andermatt, Switzerland, as shown in Figure 4.

*Case Study 1- Chiva-Som Health Resort, Hua Hin, Thailand.*

A total of 40 pictures at case study 2-MMHR, Altaussee, Austria, as shown in Figure 3, and 40 at case study 3-TCA, Gotthardstrasse, Andermatt, Switzerland, as shown in Figure 4.

*Case Study 2- MAYRLIFE Medical Health Resort, Altaussee, Austria.*

*Case Study 3- The Chedi Andermatt, Wellness Facility, Gotthardstrasse, Andermatt, Switzerland.*

The authors achieved data saturation for every case study. Photographs were clustered by checklist domain, and the stories associated with these photos were used to describe the TAEs and their effects on patients’ well-being. After collecting the photograph data, the authors presented the pictures to experts/professionals for checking and validation. Also, the design aspects of the built environment (architecture and urban planning) were analysed using photos to determine whether three facility elements are therapeutic and affect patients’ well-being and recovery. However, from the photoproduction of the three case study facilities selected that facilitate patients’ well-being and recovery, as presented in case studies 1, 2 and 3, include natural elements and biophilic design, environmental controls, spatial design and wayfinding, materials and aesthetics, and EBD as TAEs to enhance the patient well-being of the healthcare Facilities.

Table 1 shows the individual assessments of TASs across the three selected facilities: CSHR (Thailand), MMHR (Austria), and TCA (Switzerland). A scale of availability was applied, where “A” indicates presence and “N/A” indicates absence of specific features. The findings show that Chiva-Som recorded the highest availability, 27 present elements reflecting its comprehensive integration of biophilic features, lighting design, and outdoor therapy areas. MMHR is the second-best facility with 25 present elements, excelling particularly in adaptive spaces and sensory integration. In comparison, TCA had 23 present elements, showing strong use of natural materials but fewer outdoor therapeutic provisions. Overall, Table 1 highlights that while all facilities embed TASs to varying degrees, differences in emphasis, such as CSHR’s stronger biophilic integration, MMHR’s adaptive multifunctional spaces, and TCA’s material-focused approach, shape the distinct wellness experiences provided. Table 1 presents a detailed availability assessment of 30 individual TAS features across the three selected facilities. Readers are directed to Table 3 for a summarised comparative overview of scores and rankings.

Table 2 presents a granular quality assessment of individual TAS elements using a Likert scale. For a consolidated summary of cumulative scores and facility rankings, readers are directed to Table 3.

Table 2 above presents the Quality Assessment of TASs across CSHR, Thailand, MMHR Austria, TCA Switzerland, using a Likert scale with values (scores) ranging from 1 (Poor) to 5 (Excellent). CSHR, Thailand, consistently recorded the highest cumulative score (96), excelling in biophilic design, spatial organisation, and sensory integration through immersive natural landscapes, modular layouts, and multisensory therapy environments. MMHR, Austria, is the second-best, with a total of 91, reflecting strong performance in lighting design, adaptive spaces, and privacy/personalisation features, though slightly less immersive in biophilic integration than CSHR, Thailand. TCA, Switzerland, with a total of 81, emphasised luxury aesthetics, natural materials, and spa-based sensory experiences but scored relatively lower in outdoor therapeutic integration and adaptive space flexibility. Overall, the table highlights that while all facilities demonstrate a high standard of therapeutic design to varying degrees, differences in emphasis, such as CSHR’s comprehensive and immersive strategy application, MMHR’s balance of functionality with environmental integration, and TCA’s focus on materiality and ambience, rather than full therapeutic diversity, shape the distinct wellness experiences provided.

The comparative analysis of the selected case studies examines how TASs are adopted across different international facilities and how these variations influence patient recovery outcomes. By comparing CSHR Thailand, MMHR, Austria, and TCA, Switzerland, differences in strategy implementation, adoption levels, and recovery indicators become evident. This approach highlights both common patterns, such as the consistent roles of biophilic design and lighting design, and contextual differences shaped by location, culture, and facility focus. The analysis provides a balanced understanding of how TASs are applied globally, offering insights into best practices and gaps in their implementation.

Table 3 provides a consolidated comparative summary of Tables 1, 2, presenting cumulative scores, percentages, and facility rankings for rapid comparative reference.

Table 3 provides a comparative assessment of the three selected international sanatorium and wellness facilities, combining the results from Table 1 (Implemented TASs in selected facilities) and Table 2 (Quality Assessment of TASs across selected facilities). Among the three internationally reviewed therapeutic facilities, the CSHR in Hua Hin, Thailand, stood out as the top case study, earning the highest overall score of 71.93%. It demonstrates its strong alignment with EBD principles and a comprehensive integration of TASs. Close behind, MMHR in Altaussee, Austria, scored a cumulative percentage of 67.25%, securing second place overall. It reflects MMHR’s dedication to incorporating holistic health and wellness features into its design. In contrast, TCA in Gotthardstrasse, Andermatt, Switzerland, while known as a luxury wellness facility, ranked third with a notably lower cumulative percentage of 60.82%. Despite its upscale atmosphere and architectural quality, TCA’s relatively limited use of the identified TASs places it below the other two in terms of therapeutic design effectiveness.

### 4.2 TAS implementation and recovery-supportive outcomes across selected facilities

#### 4.2.1 Patient recovery indicators

Patient recovery indicators are measurable signs or outcomes used to assess how well a patient is recovering in a healthcare or therapeutic environment. These indicators reflect improvements in a patient’s physical, psychological, emotional, and functional health and can vary depending on the type of illness, treatment method, and the care environment. Below is a matched framework of TASs and key patient recovery indicators in design contexts of sanatoriums or health resorts:

Table 5 outlines the matched framework of TASs and their corresponding patient recovery indicators, assessed through observational checklists. Each strategy is directly linked to measurable outcomes that reflect its impact on patient well-being. For instance, biophilic design is associated with reduced stress, improved mood, and greater engagement with natural elements, while lighting design influences circadian rhythm regulation, sleep quality, and patient alertness. Acoustic design is linked to lower stress and fewer noise complaints, contributing to better rest and concentration, whereas spatial organisation enhances wayfinding, safety, and service delivery efficiency. Similarly, integrating outdoor spaces promotes emotional well-being and relaxation, while privacy and personalisation enhance patient comfort, satisfaction, and stress reduction. Colour psychology and material selection foster calming environments and positive visual experiences, while adaptive spaces offer flexibility for diverse therapeutic needs, ensuring inclusivity and engagement. Together, these matched frameworks illustrate how TASs correspond with observable recovery-supportive conditions, as documented in the therapeutic architecture literature. The indicators presented are literature-derived proxy measures of well-being rather than clinically validated outcome data, and should be interpreted within that context (Cho, 2023; Faraj et al., 2024).

**Table 5:** Matched framework of TASs and patient recovery indicators.

S/N	Therapeutic architectural strategies (TASs)	Patient recovery indicators (assessed via observational checklist)
1	Biophilic Design	Time spent outdoors or near greenery Reduction in stress/anxiety Improved mood Increased patient movement and engagement with nature
2	Lighting Design	Stable circadian rhythms (e.g., sleep patterns) Enhanced alertness and reduced fatigue Reduced depressive symptoms Patient comfort in day/night settings
3	Acoustic Design	Reduced noise complaints Lower stress levels Better sleep quality Higher concentration and calmness in spaces
4	Spatial Organisation	Ease of navigation (wayfinding) Decrease in staff-patient friction Efficient service delivery Safety (fewer accidents or confusion)
5	Integration of Outdoor Spaces	Patient interaction with outdoor areas Improvement in emotional well-being Reduced stay duration Observed relaxation or reflection activities
6	Privacy and Personalisation	Increased patient satisfaction Comfort in private zones (e.g., curtains, personal spaces) Reduced psychological stress Higher usage of personal areas
7	Colour Psychology and Material Selection	Calm demeanour in waiting or recovery rooms Preference for warmer/earthy tones Perceived cleanliness and serenity Positive visual stimulation
8	Adaptive Spaces	Flexible use for group/individual activities Accessibility for all abilities Engagement in therapy sessions- Reduced behavioural issues
9	Sensory Integration	Stimulated cognition or emotional balance Engagement in multisensory therapy areas Improved focus and relaxation Reduced signs of overstimulation or boredom

Source: author’s fieldwork (2025).

Table 6 presents the strategy implementation scores, derived as the cumulative sum of Implemented TASs identified in Table 1 across the selected facilities, and the patient recovery indicator scores across the three selected facilities, highlighting the relationship between architectural strategies and recovery outcomes. CSHR achieved the highest TAS implementation score of 27 and a corresponding proxy recovery indicator score of 27, reflecting its comprehensive integration of therapeutic strategies. MMHR Altaussee followed closely with an implementation score of 25 and a proxy recovery indicator score of 26, confirming the effectiveness of its patient-centred design approach. TCA recorded scores of 23 for implementation and 24 for proxy recovery indicators, suggesting that, while therapeutic elements are present, their integration is less comprehensive than at the other two facilities. Overall, the table illustrates a clear trend: higher levels of TASs adoption correspond with improved patient recovery outcomes, underscoring the importance of EBD in shaping healing environments.

**Table 6:** TASs implementation score and patient recovery indicator scores in selected facilities.

S/N	Facility	TASs implementation score	Patient recovery indicator score
1	Chiva-Som Health Resort	27	27
2	MAYRLIFE Medical Health Resort Altaussee	25	26
3	Chedi Andermatt	23	24

Source: author’s fieldwork 2025.

#### 4.2.2 Correlation analysis

Prior to presenting the correlation results, the variables and analytical approach are defined as follows. The independent variable is the TAS Implementation Score, the cumulative sum of TASs identified as present across each facility, derived from the structured observational checklist in Table 1. The dependent variable is the Proxy Recovery Indicator Score, a composite score reflecting the quality of recovery-supportive design conditions across each facility, derived from the literature-validated observational checklist in Table 6. Table 7 below presents the full correlation matrix showing the Spearman’s rank values for each TAS variable against the proxy recovery indicator scores across the three facilities.

**Table 7:** Spearman’s rank correlation matrix- TAS variables and proxy recovery indicator scores.

Variables	Spearman’s *ρ* with TAS	Association strength	Rank	*N =* 3
Natural light affects mood and well-being	0.851	Strong positive	1	3
Integration of nature/outdoor spaces	0.829	Strong positive	2	3
Adjustable lighting systems and sleep patterns	0.782	Strong positive	3	3
Layout supports comfort and recovery	0.773	Strong positive	4	3
Noise control supports relaxation	0.724	Strong positive	5	3
Colour scheme influences mood and activities	0.657	Moderate positive	6	3
Quiet/private areas aid healing process	0.638	Moderate Positive	7	3
Building materials sourced locally	0.605	Moderate positive	8	3
Adaptability of spaces affects comfort	0.591	Moderate positive	9	3
Overall TASs implementation vs. Recovery score	0.83	Strong Positive	-	3

The matrix confirms that all nine TAS variables demonstrate positive associations with proxy recovery indicator scores, with biophilic and lighting-related strategies consistently showing the strongest associations. These results are interpreted within the context of the study’s observational design and small sample size, as discussed in the Limitations section.

This study applies Spearman’s Rank Correlation Coefficient to explore the association between TAS implementation scores and proxy recovery indicator scores across three internationally recognised sanatorium and wellness facilities. Spearman’s rank correlation is appropriate for ordinal and ranked data derived from structured observational checklists and does not assume normally distributed data. It is acknowledged that, given the exploratory nature of this study and the small number of facilities assessed (*n =* 3), the correlation results are interpreted as indicative of associative trends rather than inferentially conclusive or statistically generalisable findings. The analysis is therefore applied as an exploratory tool to examine the direction and strength of association between TAS implementation and proxy recovery indicators, consistent with observational EBD research practice (Van der Zwart and Pilosof, 2020).

Table 8 presents the results of the Spearman’s rank correlation analysis between TASs and patient recovery rates. The findings reveal a strong positive association across most variables, indicating that well-implemented TASs correspond with stronger recovery-supportive design conditions. Natural light showed the strongest association (*ρ* = 0.851, rank 1), consistent with established literature linking natural light to improved mood and well-being (Cho, 2023; Faraj et al., 2024) underscoring its critical role in improving mood and overall well-being. Similarly, integration of outdoor spaces (*ρ* = 0.829, rank 2) and adjustable lighting systems (*ρ* = 0.782, rank 3) demonstrated strong associations with patient recovery, highlighting the importance of biophilic and circadian-supportive design. Layout supporting comfort and recovery (*ρ* = 0.773, rank 4) and effective noise control (*ρ* = 0.724, rank 5) also exhibited substantial positive correlations, reinforcing the impact of spatial organisation and acoustic design on healing. Privacy and personalisation through quiet areas (*ρ* = 0.638, rank 6) and colour/material choices (*ρ* = 0.657, rank 7) further contributed moderately to patient outcomes. In contrast, locally sourced building materials (*ρ* = 0.605, rank 8) and adaptable spaces (*ρ* = 0.591, rank 9) showed relatively lower but still positive correlations, suggesting that, while beneficial, these factors are less directly linked to immediate recovery outcomes than light, nature, and spatial comfort. Overall, the correlation analysis confirms that patient recovery is most strongly influenced by environmental strategies that directly affect sensory and psychological well-being.

**Table 8:** Spearman’s rank correlation between TASs and patient recovery rates.

Variables	Spearman’s *ρ* with TAS	Rank	*N =* 3
Does natural light affect mood and well-being?	0.851	1	3
Are building materials sourced from the local environment?	0.605	8	3
Does the layout support comfort and recovery?	0.773	4	3
Do quiet spaces or private areas aid in the healing process?	0.638	6	3
Facility’s noise control promotes relaxation?	0.724	5	3
Is the integration of nature (gardens/ outdoor spaces) sufficient?	0.829	2	3
Adjustable lighting systems help improve sleep patterns?	0.782	3	3
Does the adaptability of spaces (adjustable furniture) affect comfort?	0.591	9	3
The colour scheme utilised in spaces influences your mood and activities	0.657	7	3

Source: author’s fieldwork (2025).

**Figure 5:**
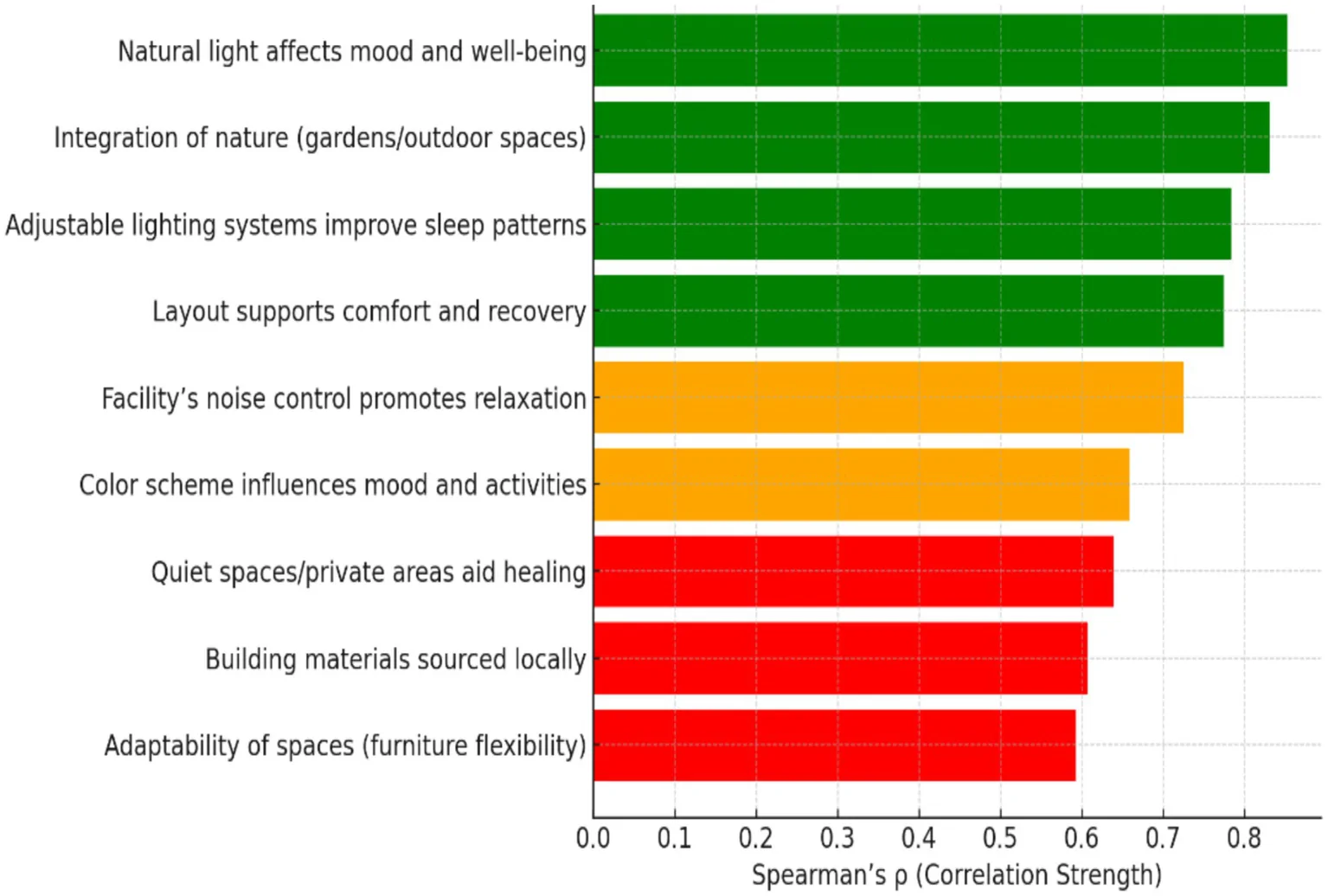
Visual representation of Spearman’s rank correlation between TASs and recovery rates. Spearman’s rank correlation heatmap.

Figure 5 shows a Spearman’s rank correlation heatmap that visually illustrates the strength of association between TASs and patient recovery rates. Darker shades indicate stronger positive correlations, while lighter shades reflect weaker relationships. The analysis reveals that natural light (*ρ* = 0.851) and integration of outdoor spaces (*ρ* = 0.829) demonstrate the strongest associations with recovery, emphasising their critical role in therapeutic environments. Adjustable lighting systems (*ρ* = 0.782) and spatial layout (*ρ* = 0.773) also show strong correlations, highlighting the importance of flexible design in supporting patient well-being. Conversely, adaptability of spaces (*ρ* = 0.591) and use of local building materials (*ρ* = 0.605) show weaker correlations, suggesting that while relevant, these factors may not directly influence patient recovery outcomes as strongly as sensory or environmental strategies. The heatmap thus provides a clear comparative perspective, reinforcing the association between biophilic and sensory-centered design interventions and positive recovery-supportive conditions in therapeutic facilities.

Table 9 shows the interpretation of Spearman’s rank. After collating and averaging the ranks for each strategy and corresponding patient recovery outcome across the three facilities, Spearman’s correlation was calculated to determine whether a statistically significant association exists. The results revealed a Spearman’s rank correlation coefficient (*ρ*) of 0.83, indicating a strong positive association between TAS implementation scores and proxy recovery indicator scores across the selected facilities. Given the small number of facilities assessed (*n =* 3), *p*-values are not reported as inferential statistics, as they cannot be meaningfully interpreted at this sample size. The findings are therefore presented as exploratory and associative, consistent with the observational design of this study. This strong positive association suggests that higher TAS implementation levels correspond with stronger proxy recovery-supportive conditions, supporting the proposition that well-designed therapeutic environments are associated with enhanced patient well-being, a finding consistent with the broader therapeutic architecture literature (Cho, 2023; Simonsen et al., 2022; Li et al., 2024). This finding confirms that facilities with higher implementation levels of TASs, such as biophilic design, spatial organisation, and privacy/personalisation, are associated with improved patient recovery outcomes. These associations are consistent with proxy indicators such as observed outdoor engagement, reported emotional well-being, and sleep quality improvements documented in the therapeutic architecture literature. The strong positive association suggests that therapeutic architecture can serve as a valuable design consideration in healthcare settings to support recovery-supportive conditions, particularly in sanatorium and wellness facilities where long-term well-being is a primary goal. These findings provide observational and associative support for systematically incorporating these strategies into future therapeutic facility designs, especially in under-optimised healthcare settings such as those commonly found in Nigeria.

**Table 9:** The interpretation of the correlation analysis.

Therapeutic strategies	r = Spearman’s rank	Interpretation
Natural light affects mood and well-being	0.851	Strong positive correlation
Integration of nature (gardens/ outdoor spaces) is sufficient	0.829	Strong positive correlation
Adjustable lighting systems help improve sleep patterns	0.782	Strong positive correlation
Layout supports comfort and recovery	0.773	Strong positive correlation
Facility noise control promotes relaxation and stress reduction	0.724	Strong positive correlation
The colour scheme utilised in spaces influences your mood and activities	0.657	Moderate positive correlation
Quiet spaces or private areas aid in the healing process	0.638	Moderate positive correlation
Building Materials are sourced from the local environment	0.605	Moderate positive correlation
Adaptability of spaces (adjustable furniture) affects comfort	0.591	Moderate positive correlation

**Figure 6:**
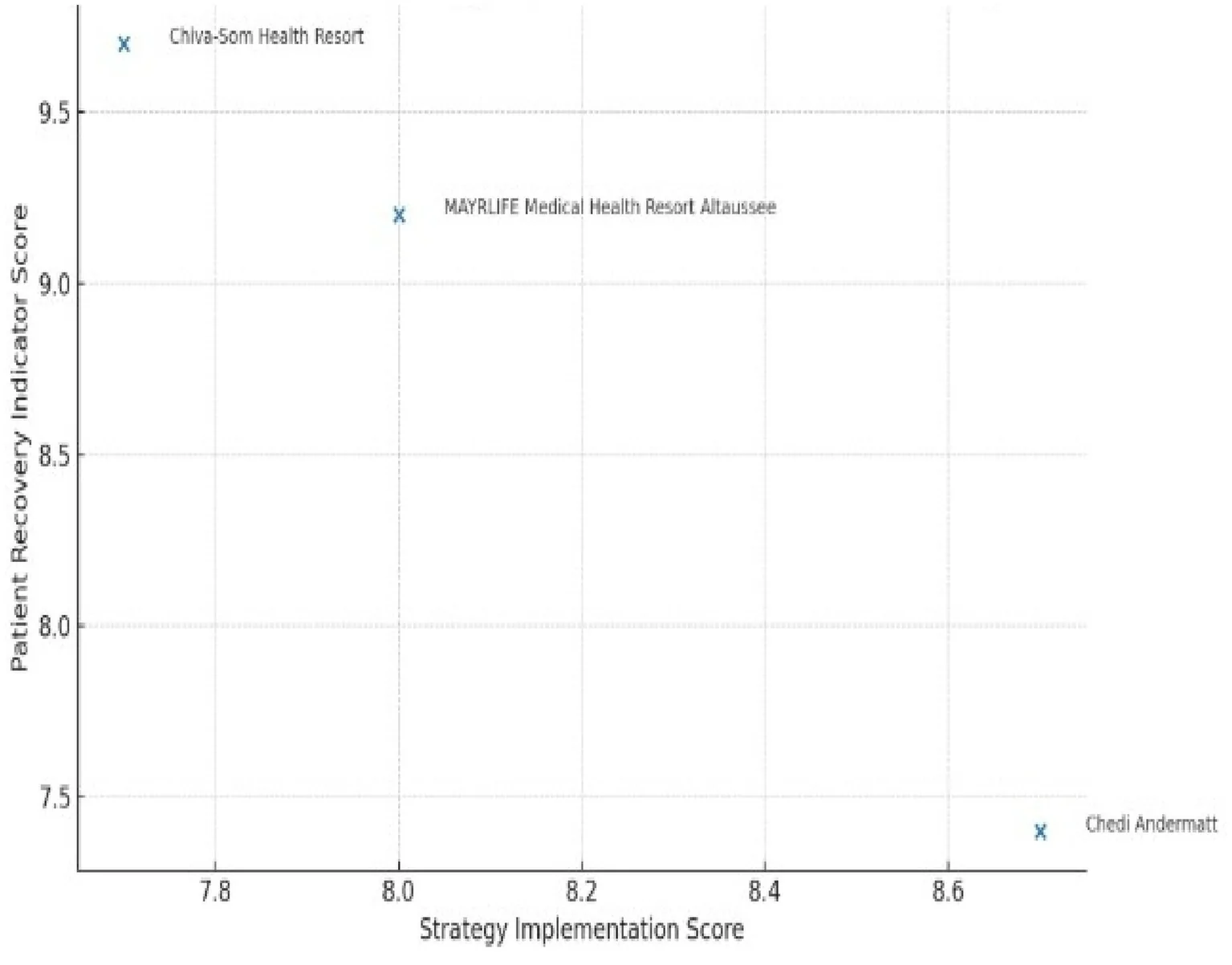
Scatter plot visualisation showing the relationship between strategy implementation score and patient recovery indicator score for the three facilities.

Figure 6 shows a scatter plot visualisation of the relationship between TASs Implementation Score and Patient Recovery Indicator Score for the three selected facilities. The upward linear trend in the data suggests a positive, monotonic relationship between the level of TAS implementation and patient recovery outcomes. It indicates that facilities with higher TAS implementation scores tend to correspond with stronger proxy recovery indicator scores, supporting the proposition that well-executed therapeutic design is associated with enhanced recovery-supportive conditions. The proximity of data points to the regression line suggests a moderately strong positive association, further explored through the Spearman rank correlation analysis presented in Table 9.

## 5 Limitations

This study acknowledges the following limitations, which should be considered when interpreting the findings and which define the scope and boundaries of the conclusions drawn.

This study does not collect primary clinical or patient-level health data. Assessment of patient recovery is based on proxy indicators derived from the existing therapeutic architecture literature (Cho, 2023; Faraj et al., 2024; Simonsen et al., 2022). The findings therefore reflect the recovery-supportive potential of the built environment rather than directly measured clinical outcomes. Future research should incorporate primary patient data and validated clinical health scales to strengthen empirical evidence.

The study examines three internationally recognised sanatorium and wellness facilities. While purposive sampling ensured information-rich case selection, the small number of facilities limits the statistical generalisability of the correlation findings. The Spearman’s rank correlation (*ρ* = 0.83) is therefore interpreted as an exploratory, indicative association rather than an inferentially conclusive finding. Larger multi-facility studies are needed to establish more robust and generalisable evidence.

Data were collected via structured observational checklists applied to publicly available architectural documentation, official facility materials, academic publications, and professional photography. While consistent with established EBD observational methods (Van der Zwart and Pilosof, 2020; Oher et al., 2024), this approach limits the depth and completeness of information obtainable. Future studies should incorporate fieldwork-based post-occupancy evaluations and direct patient engagement to strengthen data validity.

The three selected facilities are internationally recognised luxury therapeutic wellness resorts operating at the higher end of the healthcare design spectrum. While this was deliberate to examine best-practice TAS implementation within well-documented environments, it introduces positive selection bias and limits direct applicability to under-resourced or public healthcare settings, including those in Nigeria. The findings are therefore best understood as demonstrating what is achievable under high-resource conditions rather than as representative of typical healthcare environments.

The structured observational scoring system was developed from a review of peer-reviewed therapeutic architecture literature (Rebecchi et al., 2023; Li et al., 2024). While the checklist criteria are grounded in established EBD practice, formal inter-rater reliability testing was not conducted, introducing potential researcher subjectivity and limiting reproducibility. Future studies should incorporate inter-rater reliability testing and formal validation of the scoring framework against established clinical or architectural assessment tools.

Finally, this study employs an observational, non-experimental design without control variables or randomization. Causal relationships between TAS implementation and patient recovery outcomes cannot therefore be established. All findings are associative and exploratory in nature. Experimental or longitudinal research designs with matched controls would be required to move beyond association toward causal claims.

## 6 Conclusion

This study emphasises the relevance and growing necessity of integrating TASs in the design of health and wellness-oriented facilities. Through a detailed assessment of international case studies, CSHR, Thailand, MMHR, Austria, and TCA, Switzerland, the research highlights how thoughtfully planned environments contribute significantly to patient recovery and overall well-being. Key findings from the study include the following: TASs such as biophilic design, natural ventilation, daylighting, and spatial personalisation are strongly associated with conditions that support physical, psychological, and emotional recovery. Integration of outdoor spaces, water elements, and sensory zoning corresponds with enhanced patient engagement with nature and improved recovery-supportive conditions. Facilities that prioritise calm aesthetics, intuitive spatial flow, and privacy tend to record stronger proxy recovery indicator scores and higher recovery-supportive design alignment. The international case studies examined in this study serve as aspirational benchmarks demonstrating best-practice TAS implementation under high-resource conditions. Their findings offer transferable principles for adapting therapeutic design strategies to the Nigerian context, particularly in long-term care and sanatorium-type facilities where recovery-supportive design remains underexploited (Sholanke and Oke, 2020; David-mukoro et al., 2024). Challenges such as limited awareness, insufficient skilled design professionals, and inadequate policy support continue to hinder widespread TAS adoption in Nigeria and must be addressed through targeted capacity building and evidence-based design policy frameworks. Therefore, the study presents the following recommendations: Stakeholders and government bodies should develop design guidelines and policies that encourage the adoption of therapeutic principles in public and private healthcare infrastructure. Training programs and workshops should be initiated for architects, planners, and healthcare professionals to build local capacity in therapeutic design. Model therapeutic health resorts or sanatoriums should be developed in strategic locations to demonstrate the practicality and impact of these design strategies. Creating awareness among decision-makers, clients, and users on the health, emotional, and economic benefits of therapeutic design is essential. Further research should incorporate post-occupancy evaluations, primary clinical patient data, validated health outcome scales, and longitudinal research designs to move beyond observational associations toward stronger empirical evidence of TAS impact on patient recovery. Cultural adaptation of therapeutic strategies to the Nigerian healthcare context warrants particular attention. Architects, healthcare providers, psychologists, and environmental scientists must collaborate to design spaces that are healing, sustainable, and culturally appropriate.

## Data Availability

The original contributions presented in the study are included in the article/Supplementary material, further inquiries can be directed to the corresponding author.
