# Supplementary material for: Exploring therapeutic architectural strategies as recovery- supportive design interventions in selected international sanatorium and therapeutic wellness facilities
Source: Front Psychol. 2026 Jun 25;17:1830779. doi: 10.3389/fpsyg.2026.1830779 (PMC13346203; doi:10.3389/fpsyg.2026.1830779)
Supplement: Supplementary file 1 [file Data_Sheet_1.ZIP › APPENDIX II-QUESTIONNAIRE SURVEY.docx]

**APPENDIX II – QUESTIONNAIRE SURVEY**

**Validity**


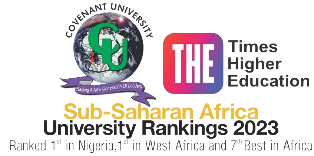


**For official use only.** No_______________ City______________

**COVENANT UNIVERSITY**

Canaan Land Km 10 Idiroko Road | P.M.B 1023, Ota, Ogun State, Nigeria.

**THERAPEUTIC ARCHITECTURAL STRATEGIES AND PATIENT RECOVERY IN THE DESIGN OF A SANATORIUM**

Dear respondents,

The design of spaces profoundly impacts health, well-being, and recovery. This study focuses on how therapeutic architectural strategies enhance patient recovery within medical facilities, particularly sanatoriums. Understanding these strategies' role in promoting healing and comfort is essential to advancing patient care. By gathering insights from medical facilities, this research aims to inform designs that prioritize therapeutic outcomes. Your participation will greatly support our efforts to bridge theory and practice in therapeutic architecture. We kindly invite you to willingly and heartily participate in this study to contribute to a transformative understanding of healthcare environments. Thank you for your support.

***Salami B. Tunmise*** *(Researcher)*

**SOCIO-DEMOGRAPHIC CHARACTERISTICS OF RESPONDENTS.**

1. Gender [ ] Male [ ] Female
2. Role [ ] Visitor [ ] Patient [ ] Staff
3. Age [ ] Below 20 [ ] 20 – 30 [ ] 31 – 40 [ ] 41 – 50 [ ] Above 50
4. Length of stay [ ] Less than 1 week [ ] 1-4 weeks [ ] 1-3 months [ ] Over 3 months
5. Reason for visit [ ] Acute Illness or injury [ ] Condition Mgt [ ] Post recovery [ ] Wellness treatment[ ] Work
6. Healthcare facility [ ] JTH [ ] Healthy Body Clinic [ ] National Hospital Abuja [ ] LASUTH

**OBJECTIVE 1: Therapeutic Architectural Strategies**

There are fundamental aspects and features of therapeutic architectural strategies that should promote a supportive and healing environment. We kindly request you to evaluate the following elements/statements related to your current space(s) in terms of their impact on well-being and recovery.

Lowest Ranking [1] [2] [3] [4] [5] Highest Ranking

Average Ranking

**OBJECTIVE 2: Architectural Design Elements**

There are essential architectural design elements that should foster a healing and supportive environment.

| **S/N** | **Therapeutic Architectural Strategies** | **1** | **2** | **3** | **4** | **5** | **Remarks** |
| --- | --- | --- | --- | --- | --- | --- | --- |
| **Strategy 1: Biophilic Design** | | | | | | | |
| 1 | Large windows |  |  |  |  |  |  |
|  | Skylights |  |  |  |  |  |  |
|  | Indoor plants |  |  |  |  |  |  |
|  | Green walls |  |  |  |  |  |  |
|  | Water Features |  |  |  |  |  |  |
|  | Views of Nature |  |  |  |  |  |  |
| TOTAL | |  | | | | |  |
| **Category 2: Lighting Design** | | | | | | | |
| 2 | Smart Lighting |  |  |  |  |  |  |
|  | Light wells |  |  |  |  |  |  |
|  | Solar Shading Devices |  |  |  |  |  |  |
| TOTAL | |  | | | | |  |
| **Strategy 3: Acoustic Elements** | | | | | | | |
| 3 | Sound-absorbing materials |  |  |  |  |  |  |
|  | Quiet zones |  |  |  |  |  |  |
|  | Noise-reducing mechanisms |  |  |  |  |  |  |
| TOTAL | |  | | | | |  |
| **Strategy 4: Spatial Organization Elements** | | | | | | | |
| 4 | Intuitive layouts |  |  |  |  |  |  |
|  | Clear Wayfinding |  |  |  |  |  |  |
|  | Centralized communal spaces |  |  |  |  |  |  |
| TOTAL | |  | | | | |  |
| **Strategy 5: Integration of Outdoor Spaces** | | | | | | | |
| 5 | Balconies |  |  |  |  |  |  |
|  | Garden |  |  |  |  |  |  |
|  | Covered walkways |  |  |  |  |  |  |
| TOTAL | | 43 | | | | |  |
| **Strategy 6: Privacy and Personalization Elements** | | | | | | | |
| 6 | Private rooms |  |  |  |  |  |  |
|  | Adjustable furnishings |  |  |  |  |  |  |
| TOTAL | |  | | | | |  |
| **Strategy 7: Color Psychology & Material Selection Elements** | | | | | | | |
| 7 | Calming color palettes, |  |  |  |  |  |  |
|  | Natural materials |  |  |  |  |  |  |
| TOTAL | |  | | | | |  |
| **Strategy 8: Adaptive Spaces elements** | | | | | | | |
| 8 | Modular furniture |  |  |  |  |  |  |
|  | Flexible layouts |  |  |  |  |  |  |
|  | Movable partitions |  |  |  |  |  |  |
| TOTAL | |  | | | | |  |
| **Strategy 9: Sensory Integration elements** | | | | | | | |
| 9 | Touch-sensitive materials, |  |  |  |  |  |  |
|  | Diffusion systems, |  |  |  |  |  |  |
|  | Scent zones |  |  |  |  |  |  |
| TOTAL | |  | | | | |  |

**OBJECTIVE 3: Impact on Patient Recovery**

The design of therapeutic spaces plays a crucial role in patient recovery. We kindly ask you to evaluate the following statements about how different design elements of your current environment have influenced your recovery process and well-being.

| **IMPACT OF THERAPEUTIC ARCHITECTURAL STRATEGIES ON PATIENT RECOVERY** | | | | | | |
| --- | --- | --- | --- | --- | --- | --- |
|  | In my medical facility | **Always (5), Very Often (4), Sometimes (3), Rarely (2), Never (1)** | | | | |
|  |  | **1** | **2** | **3** | **4** | **5** |
| 37. | Does natural light in your room affect your mood and well-being? |  |  |  |  |  |
| 38 | Do natural materials in your environment help reduce stress? |  |  |  |  |  |
| 39 | Do the layout of the facility affect your comfort and recovery? |  |  |  |  |  |
| 40 | Do quiet spaces or private areas aid in your healing process? |  |  |  |  |  |
| 41 | Does the facility’s noise control promote relaxation and recovery? |  |  |  |  |  |
| 42 | Does the integration of nature (gardens/ outdoor spaces) support your recovery? |  |  |  |  |  |
| 43 | Does adjustable lighting improve your sleep and recovery? |  |  |  |  |  |
| 44 | Does the adaptability of spaces (adjustable furniture) affect your comfort? |  |  |  |  |  |
| 45 | Does the facility’s color scheme or design influence your mood and recovery? |  |  |  |  |  |
| 46 | How satisfied are you with the overall design in supporting your recovery? |  |  |  |  |  |
