# Supplementary material for: Exploring therapeutic architectural strategies as recovery- supportive design interventions in selected international sanatorium and therapeutic wellness facilities
Source: Front Psychol. 2026 Jun 25;17:1830779. doi: 10.3389/fpsyg.2026.1830779 (PMC13346203; doi:10.3389/fpsyg.2026.1830779)
Supplement: Supplementary file 1 [file Data_Sheet_1.ZIP › APPENDIX I-OBSERVATION GUIDE.docx]

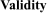

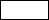

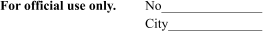
**APPENDIX I** **- OBSERVATION GUIDE**


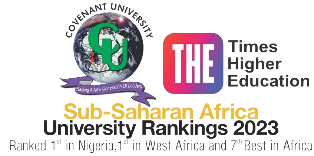


**COVENANT UNIVERSITY** Canaan Land Km 10 Idiroko Road | PMB 1023, Ota, Ogun State, Nigeria.

**THERAPEUTIC ARCHITECTURAL STRATEGIES AND PATIENT RECOVERY IN THE DESIGN OF A STATE SANATORIUM**

1. **Therapeutic Architectural Strategies**

| **S/N** | **Building Features** | | **A** | **N/A** | **Remarks** |
| --- | --- | --- | --- | --- | --- |
| 1 | Biophilic Design | Indoor Gardens and Green Spaces |  |  |  |
|  |  | Water Features |  |  |  |
|  |  | Healing Gardens |  |  |  |
|  |  | Rooftop Terraces and Green Roofs |  |  |  |
|  |  | Healing Lounges and Social Hubs |  |  |  |
| 2 | Lighting Design | Natural Lighting |  |  |  |
|  |  | Therapeutic Colored Lighting |  |  |  |
|  |  | Dimmable Lighting Controls |  |  |  |
| 3 | Acoustic Design | Library and Reading Rooms |  |  |  |
|  |  | Spiritual and Reflection Rooms |  |  |  |
|  |  | Meditation and Relaxation areas |  |  |  |
| 4 | Spatial Organization | Signages |  |  |  |
|  |  | Pavilions and Gazebo |  |  |  |
|  |  | Dining and Nutrition Spaces |  |  |  |
|  |  | Outdoor Seating Zones |  |  |  |
| 5 | Integration of Outdoor Spaces | Outdoor Therapy Areas |  |  |  |
|  |  | Open-Air Yoga Areas |  |  |  |
|  |  | Walking Trails and Jogging Paths |  |  |  |
| 6 | Privacy and Personalization | Patient Rooms |  |  |  |
|  |  | Hydrotherapy Rooms |  |  |  |
|  |  | Therapy Rooms |  |  |  |
|  |  | Rehabilitation Gym |  |  |  |
| 7 | Color Psychology and Material Selection | Soothing Colors- Blues/Green/ Earthy Tones |  |  |  |
|  |  | Warm Colors- Muted Oranges & Yellows |  |  |  |
|  |  | Neutral and Pastel shades- Off-whites, beiges, pastels |  |  |  |
|  |  | Natural Material- Wood, Stone, Bamoo |  |  |  |
| 8 | Adaptive Spaces | Multi-Functional Rooms |  |  |  |
|  |  | Nature-Integrated Areas |  |  |  |
| 9 | Sensory Integration | Aromatherapy |  |  |  |
|  |  | Soft Furnishings |  |  |  |
|  |  | Sensory Rooms |  |  |  |
|  |  | TOTAL |  |  |  |
