# Supplementary material for: Exploring therapeutic architectural strategies as recovery- supportive design interventions in selected international sanatorium and therapeutic wellness facilities
Source: Front Psychol. 2026 Jun 25;17:1830779. doi: 10.3389/fpsyg.2026.1830779 (PMC13346203; doi:10.3389/fpsyg.2026.1830779)
Supplement: Supplementary file 1 [file Data_Sheet_1.ZIP › APPENDIX IV-INFORMED CONSENT FORM.docx]

**APPENDIX IV - INFORMED CONSENT FORM**


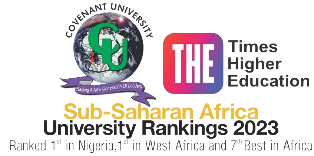


Good morning/afternoon. I am a researcher and principal researcher researching **“Measuring the Impact of Therapeutic Architectural Strategies (TASs) on Patient Recovery in Selected Sanatorium Facilities”** towards a research project to be published. I am here to ask you some questions on Therapeutic architectural strategies (TASs), patient recovery, sanatorium design, holistic care, therapeutic facilities, Therapeutic architectural elements (TAEs), evidence-based design (EBD), TAEs in healthcare Facilities that Enhance Patients’ Well-Being, impact of TASs on Patient recovery, and Patient Recovery Indicators. Thank you for choosing to take part in this study. I will be interviewing you today. Before we start the interview, I will briefly explain the exercise and answer any questions you may have.

**The Aims of the Study**

This article investigates the impact of TASs on patient recovery within sanatoriums by identifying key TAEs that impact patient recovery in selected healthcare facilities. This study may help architects and healthcare providers to design facilities with elements like access to nature, optimised lighting and acoustics, and comfortable social spaces to foster well-being, enhance recovery rates, and improve the overall quality of care.

**Data Collection**

Data collection instruments employed for this study were an observational checklist developed based on a review of literature on TAS, a qualitative photograph checklist used for qualitative data, and a questionnaire for quantitative data. The checklist focused on nine key TASs.

**Confidentiality**

This information will be for academic purposes only. I am asking for your help to ensure the information I collect is accurate. You may refuse to fill in the questionnaire or choose to stop at any time. Also, be assured that we will treat your response with strict confidentiality.

I will now complete a form with some background information about you. It will allow us to get some context about the results of this study. Thank you. We are now ready to start the interview.

Do you have any questions about this exercise?

Do I have your agreement to proceed?

Respondent’s signature and date

……………………………………………….

(Indicates the respondent’s willingness to participate)
