# Supplementary figures and images for: Exploring therapeutic architectural strategies as recovery- supportive design interventions in selected international sanatorium and therapeutic wellness facilities
Source: Front Psychol. 2026 Jun 25;17:1830779. doi: 10.3389/fpsyg.2026.1830779 (PMC13346203; doi:10.3389/fpsyg.2026.1830779)

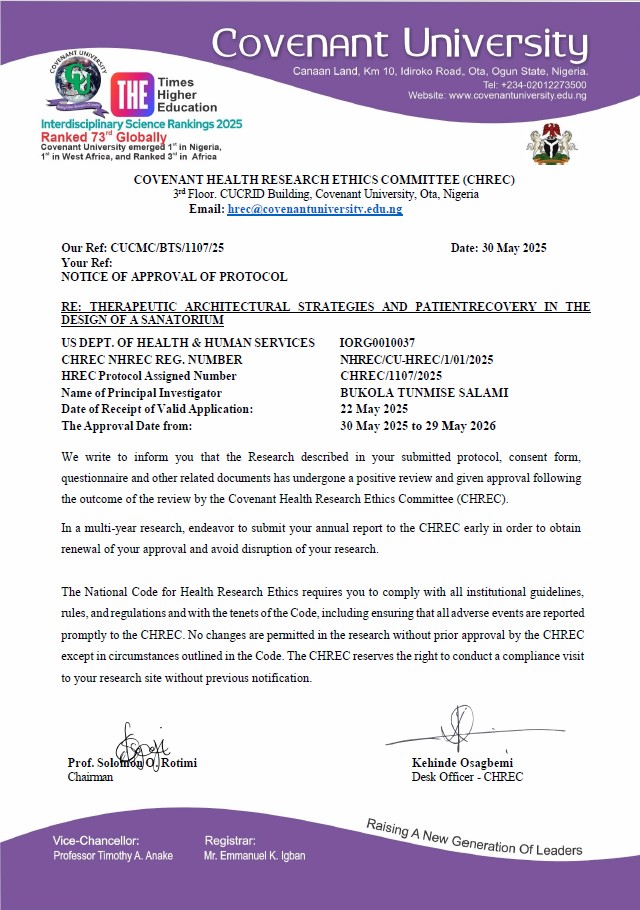

Supplement: Supplementary file 1 [file Data_Sheet_1.ZIP › APPENDIX III-ETHICAL APPROVAL CERTIFICATE.docx]
